# *Stromal Cell-Derived Factor 1* Polymorphism in Retinal Vein Occlusion

**DOI:** 10.1371/journal.pone.0166544

**Published:** 2016-11-10

**Authors:** Andrea Szigeti, Mónika Ecsedy, Miklós Schneider, Lilla Lénárt, Balázs Lesch, Zoltán Zsolt Nagy, Andrea Fekete, Zsuzsanna Récsán

**Affiliations:** 1 Department of Ophthalmology, Semmelweis University, Budapest, Hungary; 2 MTA-SE Lendület Diabetes Research Group, Research Laboratory for Pediatrics and Nephrology of the Hungarian Academy of Sciences and of the Semmelweis University, Budapest, Hungary; University of Birmingham, UNITED KINGDOM

## Abstract

**Background:**

*Stromal cell-derived factor 1* (*SDF1*) has crucial role in the regulation of angiogenesis and ocular neovascularisation (NV). The purpose of this study was to evaluate the association between *SDF1*-3’G(801)A polymorphism and NV complications of retinal vein occlusion (RVO).

**Methods:**

130 patients with RVO (median age: 69.0, range 35–93 years; male/female– 58/72; 55 patients had central RVO, 75 patients had branch RVO) were enrolled in this study. In the RVO group, 40 (30.8%) patients were diagnosed with NV complications of RVO and 90 (69.2%) patients without NVs. The median follow up period was 40.3 months (range: 18–57 months). The *SDF1*-3’G(801)A polymorphism was detected by PCR-RFLP. Allelic prevalence was related to reference values obtained in the control group consisted of 125 randomly selected, age and gender matched, unrelated volunteers (median age: 68.0, range 36–95 years; male/female– 53/72). Statistical analysis of the allele and genotype differences between groups (RVO patients vs controls; RVO patients with NV vs RVO patients without NV) was determined by chi-squared test. P value of <0.05 was considered statistically significant.

**Results:**

Hardy-Weinberg criteria was fulfilled in all groups. The *SDF1-*3’G(801)A allele and genotype frequencies of RVO patients were similar to controls (*SDF1*-3’A allele: 22.3% vs 20.8%; *SDF1*-3’(801)AA: 5.4% vs 4.8%, *SDF1*-3’(801)GG: 60.8% vs 63.2%). The frequency of *SDF1*-3’(801)AA and *SDF1*-3’(801)GA genotypes, as well as the *SDF1-*3’(801)A allele frequency were higher in RVO patients with NV versus in patients without NV complication (*SDF1*-3’(801)AA+AG genotypes: 57.5% vs 31.1%, p = 0.008; *SDF1*-3’(801)A allele: 35.0% vs 16.7%, p = 0.002) or versus controls (*SDF1*-3’(801)AA+AG genotypes 57.5% vs 36.8%, p = 0.021; *SDF1*-3’(801)A allele: 35.0% vs 20.8% p = 0.01). Carrying of *SDF1*-3’(801)A allele increased the risk of neovascularisation complications of RVO by 2.69 (OR, 95% CI = 1.47–4.93).

**Conclusion:**

These findings suggest that carrying *SDF1*-3’(801)A allele plays a role in the development of neovascular complications in retinal vein occlusion.

## Introduction

Retinal vein occlusion (RVO) is the most common form of retinal vascular disease following diabetic retinopathy with a prevalence rate of 5.2/1000, and may result in permanent vision loss. [1–2]

Beside macular edema, the most serious complication of RVO is neovascularisation (NV), causing vitreous haemorrhage, neovacular glaucoma and fibrovascular membranes with consecutive tractional retinal detachment, which further impairs vision. [3–5] The greatest risk of developing anterior segment NV is during the first 7–8 months after the event, then the risk dramatically falls to minimal. [3,6] In a recent review of central retinal vein occlusion (CRVO) cases the NV incidence was around 50% at 6 months after the occlusion where the ischemic subtype was not defined, while in ischemic CRVO the incidences of iris, angle NV and neovascular glaucoma were 57.7%, 47.4% and 33.3%. [5, 7]

Incidence of NV in branch retinal vein occlusion (BRVO) is difficult to estimate, retinal NV develops in 22–31% of the patients with BRVO. [8–10] NVs occur mostly at the border of perfused and non-perfused retina, while NV at the disc and iris is rare. [3–4] The risk of NV increases when the area of capillary nonperfusion exceeds 5 disc diameters.[10]

Stromal cell-derived factor 1 (*SDF1*) also known as CXCL12 is a highly active small (68-amino-acid, 8 kDa) chemokine, which is responsible for chemotaxis of CXCR4 expressing cells including CD34^+^ hematopoietic stem cells (HSCs), monocytes, lymphocytes, and endothelial progenitor cells (EPCs). Through complex interactions with adhesion molecules, *SDF1* promotes transendothelial migration and attachment of CD34^+^ HSCs to the vascular endothelium. [11–13] The existence of a regulatory loop between VEGF-A and *SDF1* supports the crucial role of the *SDF1* in the regulation of angiogenesis and formation of the new blood vessels in the eye. [10, 14] Higher intravitreous *SDF1* protein level has been measured in RVO patients with NV when compared to patients without NV, or negative controls, suggesting a pivotal role of *SDF1* in the development of neovascular changes during RVO. [15]

*SDF1*-3’G(801)A polymorphism is the most studied single nucleotide polymorphism in *CXCL12* gene which encodes for SDF1 [16]. The *SDF1*-3’G(801)A allele is the possible target of cis-acting factors enhancing the expression of *CXCL12* gene and the stability of the mRNA transcript. Furthermore *SDF1-3’*(801)A allele carrying homozygotes (*SDF1-3’*(801)AA) have higher SDF1 protein level, than *SDF1-3’*(801)A allele carrying heterozygotes (*SDF1-3’*(801)AG) or wild *SDF1*-3’(801)G allele carrying homozygotes (*SDF1-3’*(801)GG). [17–18].

Since the relevance of *SDF1* in retinal neovascularisation has been already highlighted and the *SDF1*-3’G*(801)*A polymorphism has been shown to increase the production of *SDF1* the purpose of this study was to evaluate the potential association between *SDF1*-3′G(801)A polymorphism and NV complications of RVO.

## Patients and Methods

This prospective controlled study was performed at the Department of Ophthalmology and at the Laboratory for Genetics, 1st Department of Pediatrics, Semmelweis University, Budapest, Hungary. All participants were treated in accordance with the tenets of the Declaration of Helsinki. Institutional Review Board approval was obtained for all study protocols (Semmelweis University Regional and Institutional Committee of Sciences and Research Ethics). Written informed consent was obtained from all participants in this study.

*Inclusion criteria*: Patients with retinal vein occlusion diagnosed at the Retinal and Macular Diseases outpatient unit of the Department of Ophthalmology, Semmelweis University between January 2010 and January 2013 and commitment to at least 18 months’ follow-up.

*Exclusion criteria*: Patients with a history of previous intraocular surgery, eye trauma, any other retinal or neurological disease (e.g. multiple sclerosis), intraocular inflammation or tumor were excluded from the study.

The control group consisted of 125 randomly selected age and gender matched, unrelated volunteers (median age: 68.0 years, range: 36–95 years, male/female– 53/72), who were referred for spectacle prescription, general routine ophthalmic follow-up (because of diabetes mellitus or hypertension) or for driver’s license permit renewal. All cases and controls were Caucasians. Inclusion and exclusion criteria were the same for the RVO group and the age and gender matched control group except for the fact that patients in the control group had no RVO.

*Clinical data*: RVO was classified according to the anatomical location of the occlusion. BRVO was diagnosed as “major” when one of the major branch veins draining one of the retinal quadrants was occluded. “Macular” BRVO diagnosis was used to describe cases when occlusion is limited to a small vessel draining a sector of the macular region. [19]

Slit-lamp biomicroscopy, intraocular pressure measurement with applanation tonometry, indirect ophthalmoscopy following pupil dilation, fundus fluorescein agiography, gonioscopy were used to identify eyes with NV complications of RVO during the follow up period. If the iris neovascularisation was questionable, we performed anterior segment fluorescein angiograpy. If there was any suspicion for posterior segment neovascularisation, retinal fluorescein angiography was performed. All patients with RVO also underwent systemic examinations, including fasting blood glucose level determination, systemic blood pressure measurement and detailed cardiovascular and hematological examination.

### *SDF1*-3’G(801)A genotyping

Blood samples taken for regular routine checkup were used for genotyping. Genomic DNA was extracted from whole blood by using the QIAamp Blood Mini kit (Qiagen, Hilden, Germany) according to the manufacturer’s protocol. The extracted DNA samples were then stored at −20°C for further use.

**Fig 1:**
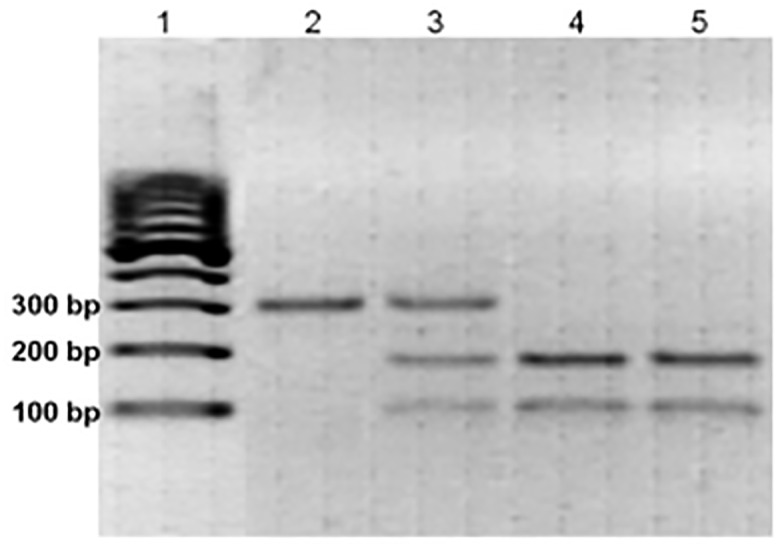
Representative sample of the genotyping for *SDF1*-3’G(801)A by PCR-RFLP. *Msp I* digestion of the PCR product of *SDF1* gene. DNA molecular weight marker is shown in *lane 1*. *Lane 2* represents sample at 302 bp denoting *SDF1*–3’homozygous (801)AA mutant genotype, *lane 3* shows bands at 302, 202 and 100 bp denoting heterozygous mutant *SDF1*-3’(801)GA and *lanes 4 and 5* at 202 and 100 bp denoting the wild type *SDF1*-3’(801)GG genotype.

The *SDF1*-3’G(801)A polymorphism was detected with polymerase chain reaction-restriction fragment length polymorphism (PCR-RFLP) method by *MspI* (HpaII) restriction enzyme (Sigma Chemical Co., Budapest, Hungary) digestion from the fragment of 302 bp DNA, previously amplified with the following primers: forward: 5’CAGTCAACCTGGGCAAAGCC3’ and reverse: 5’AGCTTTGGTCCTGAGAGTCC 3’). The following program was used for fragment amplification: one cycle of 94°C for 5 min, 94°C for 1min (denaturation), 1min at 50°C for annealing, 72°C for 1 min (elongation) followed by 34 cycles of 94°C for 1min, 50°C for 1 min, and 72°C for 1 min. The cleaved PCR products were electrophoresed on 3% agarose gel stained with ethidium bromide. Samples exhibiting only 302-bp band were assigned as *SDF1*-3’(801)AA genotype, and samples revealing two bands of 202 and 100 bp were typed as SDF1-3’(801)GG genotype, and samples illustrating three bands of 302, 202, and 100 bp were assigned as SDF1-3’(801)GA genotype. (Fig 1)

### Statistical Analysis

Statistical analyses were performed using SPSS software program (Statistical Package for Social Sciences, SPSS version 22.0; SPSS Inc., Chicago, IL, USA).

Differences between demographic data of RVO patients and control group were assessed by Chi-square test for categorical variables (gender, presence of hypertension and diabetes mellitus). The distribution of the age of patients with RVO and control patients were tested by Kolmogorov-Smirnov and Shapiro-Wilk tests and since the data was not normally distributed we chose Mann-Whitney U nonparametric test for the comparison of the age in different groups. Allele and genotype frequencies were calculated in patients and healthy controls by direct counting.

Evaluation of the Hardy-Weinberg equilibrium (HWE) was performed by Arlequin software (version 3.5.2.2; Computational and Molecular Population Genetics Lab, Bern, Switzerland) for both the polymorphisms in RVO patients and controls by comparing the observed and expected frequencies of the genotypes using chi-squared analysis.

Statistical analysis of the allel and genotype differences between groups was determined by chi-squared test, and if the expected frequencies were 5 or less, we used Fisher’s exact probability test. Risks were examined by odds ratios (OR) and 95% confidence intervals (95% CI) were calculated with the corresponding Chi-square distribution test. P values <0.05 were considered as significant.

## Results

### Clinical data

After the exclusion of subjects with less than 18 months of follow-up we had 130 patients with RVO (median age: 69.0 years, range: 35–93 years, male/female– 58/72) in this study. Fifty-five (42.3%) patients had CRVO, 75 (57.7%) patients had BRVO. In the BRVO group 54 patients (72.0%) had major BRVO and 21 patients (28.0%) had macular BRVO. Fifty-three patients (70.7%) had superotemporal BRVO and 22 (29.3%) had inferotemporal BRVO in the BRVO group. Right eye was affected in 63 patients (48.5%) and left eye in 67 patients (51.5%). The mean follow-up of RVO patients was 40.3 months (range: 18–57 months). During the follow up period, 40 (30.8%) patients (24 in CRVO group, 16 in major BRVO group) developed NV in the RVO group. The 24 patients with CRVO developed anterior segment NV, 7 of them were diagnosed with neovascular glaucoma, 16 of the BRVO patients were diagnosed with retinal NV.

The characteristics of the patients and controls are summarized in Table 1. No significant differences were found between the RVO patients and the control group and RVO patients with and without NV in main clinical parameters including age, gender and main general risk factors (diabetes mellitus, hypertension).

**Table 1:** Patients and controls characteristics.

**Variables**	**RVO patients**	**Control patients**	**P values**
**Number**	130 (55 CRVO, 75 BRVO)	125	
**Gender (male/female)**	58/72	53/72	0.818^†^
**Age (median, range, years)**	69.0 (35–93)	68.0 (36–95)	0.712^††^
**Hypertension (n, %)**	70 (53.8%)	66 (52.8%)	0.967^†^
**Diabetes mellitus (n, %)**	25 (19.2%)	31 (24.8%)	0.356^†^
**Variables**	**RVO patients without NV**	**RVO patients with NV**	**P values**
**Number**	90 (31 CRVO, 59 BRVO)	40 (24 CRVO, 16 BRVO)	
**Gender (male/female)**	38/52	20/20	0.527^†^
**Age (median, range, years)**	67.5 (35–90)	70.0 (37–93)	0.226^††^
**Hypertension (n, %)**	43 (47.8%)	27 (67.5%)	0.059^†^
**Diabetes mellitus (n, %)**	14 (15.6%)	11 (27.5%)	0.176^†^

Chi-squared test for categorical variables (^†^) and Mann-Whitney U test for continuous variable (^††^).

### Genotype frequencies and allele distribution

Genotype frequencies fulfilled the Hardy-Weinberg equilibrium criteria in all groups (p = 0.967 for all participants, p = 0.930 for controls, p = 1.0 for RVO patients).

The *SDF1* 3’G(801)A allele and genotype frequencies of RVO patients were similar to controls. (p>0.05, see Table 2.)

**Table 2:** *SDF1-* 3’G(801)A allele and genotype frequencies in control and RVO group and p values of significance regarding the comparison between the RVO patients and the controls.

**Alleles**	**A allele (n, %)**	**G allele (n, %)**	**P-value**
RVO patients	58 (22.3%)	202 (77.7%)	
Control patients	52 (20.8%)	198 (79.2%)	0.759
**Genotypes**	**AA**	**AG+GG**	**P-value**
RVO patients	7 (5.4%)	123 (94.6%)	
Control patients	6 (4.8%)	119 (95.2%)	0.832
**Genotypes**	**AA+AG**	**GG**	**P-value**
RVO patients	51 (39.2%)	79 (60.8%)	
Control patients	46 (36.8%)	79 (63.2%)	0.787

*SDF-1* 3’G(801)A allele and genotype frequencies in RVO patients with (n = 40) or without NV (n = 90) are summarized in Table 3.

**Table 3:** *SDF1*-3’G(801)A allele and genotype frequencies in RVO patients with (n = 40) or without NV (n = 90) and p values of significance regarding the comparison between the two groups of RVO patients.

**Alleles**	**A allele (n, %)**	**G allele (n, %)**	**P**	**OR (95% CI)**
RVO patients with NV	28 (35.0%)	52 (65.0%)	**0.002**	2.69 (1.47–4.93)
RVO patients without NV	30 (16.7%)	150 (83.3%)		
**Genotypes**	**AA**	**AG+GG**	**P**	**OR (95% CI)**
RVO patients with NV	5 (12.5%)	35 (87.5%)	**0.028**^‡^	6.29 (1.17–33.93)
RVO patients without NV	2 (2.2%)	88 (97.8%)		
**Genotypes**	**AA+AG**	**GG**	**P**	**OR (95% CI)**
RVO patients with NV	23 (57.5%)	17 (42.5%)	**0.008**	3.00 (1.39–6.47)
RVO patients without NV	28 (31.1%)	62 (68.9%)		

^‡^Fisher’s exact test

In the group of RVO patients diagnosed with NV complications, both the frequency of the *SDF1*-3’(801)A allele, as well as the *SDF1*-3’(801)AA genotype were higher than in RVO patients without NV complications. *SDF1-*3’(801)A allele (35.0% vs 16.7%, p = 0.002) and *SDF1-*3’(801)AA genotype (12.5% vs 2.2%, p = 0.028) increased the risk of NV complications (A allele: 2.69 [OR; 95% CI = 1.47–4.93]; AA genotype 6.29 [OR; 95% CI = 1.17–33.93]

The frequency of *SDF1*-3’(801)AA and *SDF1*-3’(801)AG genotypes in RVO group with NV was higher than in the group of RVO patients without NV complications too. (57.5% vs 31.1%, p = 0.008) *SDF1*-3’(801)AA and (801)AG genotype was found to increase the risk of NV complications by 3.00 [OR; 95% CI = 1.39–6.47]

Neither the allele nor the genotype frequencies were different in RVO patients without NV when compared to controls (*SDF1*-3’(801)A allele: 16.7% vs 20.8%, p = 0.282; *SDF1*-3’(801)AA genotypes: 2.2% vs 4.8% p = 0.473; *SDF1*-3’(801)AA+AG genotypes 31.1% vs 36.8%, p = 0.386). However in RVO patients diagnosed with NV complications, both the frequency of the *SDF1-*3’(801)A allele and *SDF1*-3’(801)A carrying genotypes (*SDF1*-3’(801)A allele: 35.0% vs 20.8%, p = 0.01; *SDF1*-3’(801)AA+AG genotypes 57.5% vs 36.8%, p = 0.021) was higher than in control group.

## Discussion

According to our knowledge this is the first investigation focusing on the relevance of *SDF1*-3′G(801)A polymorphism in patients with retinal vein occlusion. We found significant association between the development of neovascular complications and the presence of the *SDF1-*3′G (801)A allele.

Neovascular complications usually occur in the first 7–8 months in CRVO and 6–12 months in BRVO [3–7] In order to minimize the early drop-out of patients we only enrolled those participants whose follow-up was ensured for at least 18 months.

In this present study the frequency of the *SDF1*-3’(801)A allele in RVO patients with NV complications was higher than in RVO patients without NV complications resulting in an almost tripled risk increase. The frequencies of homozygous *SDF1*-3’(801)AA and *SDF1*-3’(801)GA heterozygous genotypes were also higher in RVO patients with NV complications than in patients without NV and controls. These findings suggest that carrying of *SDF1*-3’(801)A allele predisposes for the development of NV complications in patients with RVO.

*SDF1* is essential for the migration, homing and differentiation of circulating HSCs and EPCs during the retinal neovascularisation process. [11–12, 20–21] In experimental models, *SDF1* was found to be expressed and upregulated in an ischemia model of the retina in rats. [22–23] *SDF1* induced CXCR-4 (seven-transmembrane domain G-protein coupled receptor) activation leads to β-integrin expression and the binding of vascular cell adhesion molecule (VCAM)-1 to endothelial cells. [24–25] In retinal endothelial cells increased *SDF1* expression resulted in a higher VCAM-1 production [12], which plays an important role in HSCs homing and mobilization. [12, 26] It has been also demonstrated that *SDF1* had an effect on retinal endothelial cells and on the occludin gap junction protein, which is responsible for the tight junctions between endothelial cells to prevent leakage of the vessel. The retinal endothelial expression of occludin is decreased with increasing *SDF1* level. [11–12]

Only a few previous studies have investigated the vitreous level of *SDF1* in ocular diseases with neovascularisation complications. [15, 27–29] Chen et al found higher *SDF1* levels in patients with proliferative diabetic retinopathy than in non-diabetic controls. Furthermore in proliferative diabetic retinopathy patients the vitreous concentration of *SDF1* correlated well with the VEGF level. [27] In another study using a murine model of proliferative adult retinopathy exogenous *SDF1* promoted neovascularization further [11–12]. Vitreous *SDF1* alpha level was also increased in eyes with vascularly active stage 4 retinopathy of prematurity (ROP) versus either inactive stage 4 ROP or control eyes. [29]

In RVO patients higher intravitreous *SDF1* levels were measured in the presence of NV complications compared to RVO patients without NV complications or controls. [15]

All these data suggest that *SDF1* is a prominent contributor in the progression of retinal neovascularisation with a special emphasis on the angiogenic changes during RVO. [15] Higher VCAM-1 production and decreased occludin levels may be involved in the break-down of the blood-retinal barrier in RVO. SDF1 could promote neovascularisation by causing a disruption of the blood-retinal barrier through interactions with VCAM-1, β-integrin and occludin.

*SDF1* gene (also known as CXCL12 gene) is located on human chromosome 10q11.1. In the single nucleotide polymorphism of *SDF1*-3’G(801)A is mutated to A at position 801 in 30-untranslated region in the b transcriptional splice variant. [16–17]

The allelic frequency of *SDF1*-3’G(801)A was reported to vary between 0.16–0.21 in Caucasian population which is in line with the prevalence of *SDF1*-3’(801)AA and AG genotypes in our study population. [30–33] Gu XL et al.[18] recently described that both mRNA expression and protein level of SDF1 is increased in the monocytes of healthy subjects with *SDF1*–3’(801)AA than those with GA or GG genotypes.

Djuric Z et al. found that the *SDF1*-3’(801)AA genotype is more frequent in patients with proliferative diabetic retinopathy and pointed to a possible role of this allelic variant in the development of proliferative diabetic retinopathy. [30]

The incidence of diabetes mellitus in our RVO patients was 19.2%, in previous studies it ranged from 14% to 34% [6, 34] According to a previous study by Dubois-Laforgue et al. [33] SDF1-3’(801)A polymorphism was similar in Caucasian patients with or without diabetes mellitus.

The effect of diabetes mellitus on the risk of developing NV in RVO is controversial. Previous studies found higher rates of NV in diabetic patients with CRVO than in non-diabetic patients with CRVO. [35–36] Others reported no significant association between diabetes and neovascular complications in retinal vein occlusion. [7]

We did not exclude diabetic patients from our study only those patients who had diabetic retinopathy that could mimic RVO and induce neovascular proliferation. We did not find significant difference in the presence of diabetes mellitus among the RVO patients with and without NV in our study. In order to include the persence of diabetes mellitus as possible confounding systemic risk factor in the statistical analysis, a larger sample size would be needed. This is a limitation of our study.

None of our BRVO patients did develop neovacular glaucoma. It is in concordance with observation published by Hayreh et al. They found that in BRVO the retina and optic disc were the major sites of NV and none of their BRVO eyes (191 major BRVO, 74 macular BRVO eyes) did develop neovascular glaucoma. [3]

None of our patients with CRVO did develop retinal NV. This finding is in concordance with the observation published by Hayreh et al. that in CRVO the chance for the development of anterior segment NV is much greater than for posterior segment NV. [7] In this study, we investigated the association between SDF1-3′G(801)A polymorphism and presence of SDF1-3’G(801)A allele and NV complications in patients with retinal vein occlusion for the first time. Due to our relatively small sample size, we did not evaluate the polymorphism in subgroups of different types of RVO. Our preliminary results should be confirmed in a larger cohort with subgroup analysis.

There are certain limitations to our study. First, it should be noted that all of our study participants were Hungarian white Caucasians, and thus, the possibility of ethnicity as a confining factor could be excluded. Second, here we evaluated hypertension and diabetes mellitus as possible risk factors. Other systemic risk factors such as hyperlipidemia, cardiovascular diseases, blood hyperviscosity and diseases with hypercoagulation were not included. Further data collection is in progress to analyze these values as possible limiting factors. Third, due to our relatively small sample size, these are rather preliminary results which should be confirmed in larger cohorts and in subgroups of different types of RVO as well.

### Conclusion

In summary, this is the first study evaluating *SDF1*-3’G(801)A polymorphism in patients with retinal vein occlusion. We found significantly higher frequencies of the *SDF1-*3’(801)A allele and *SDF1*-3’(801)AA and *SDF1*-3’(801)GA genotypes in RVO patients with NV compared to patients without NV and to controls. Since *SDF1*-3’G(801)A polymorphism has been shown to increase the production of *SDF1* protein, which could promote neovascularisation by causing a disruption of the blood-retinal barrier, presence of *SDF1-*3’(801)A allele may be genetic risk factors for NV complications after RVO.

## Supporting Information

S1 Table Demographical characteristics (age, gender, main general risk factors: diabetes mellitus, hypertension, follow-up time, type of RVO) and *SDF-1* 3’G(801)A polymorphism data of RVO patients. (XLS)

S2 Table Demographical characteristics (age, gender, presence of diabetes mellitus, hypertension) and *SDF-1* 3’G(801)A polymorphism data of our study control subjects. (XLS)

## Abbreviations

RVO: retinal vein occlusion
BRVO: branch retinal vein occlusion
CRVO: central retinal vein occlusion
SDF1: stromal cell-derived factor 1
HSCs: hematopoietic stem cells
EPCs: endothelial progenitor cells
NV: neovasularisation
PCR-RFLP: polymerase chain reaction-restriction fragment length polymorphism
VCAM-1: vascular cell adhesion molecule-1
OR: odds ratio
CI: confidence interval
Bp: base pairs
VEGF: vascular endothelial growth factor
ROP: retinopathy of prematurity

## References

[1] Browning DJ. Retinal Vein Occlusion: Evidence-based management. 1st ed New York: Springer Science Buisness Media; 2012.

[2] Rogers S, McIntosh RL, Cheung N, Lim L, Wang JJ, Mitchell P, et al International Eye Disease Consortium. The prevalence of retinal vein occlusion: pooled data from population studies from the United States, Europe, Asia, and Australia. Ophthalmology. 2010;117: 313–9.e1. 10.1016/j.ophtha.2009.07.017 20022117 PMC2945292

[3] Hayreh SS, Rojas P, Podhajsky P, Montague P, Woolson RF. Ocular neovascularization with retinal vascular occlusion-III. Incidence of ocular neovascularization with retinal vein occlusion. Ophthalmology. 1983;90: 488–506. 6192376 10.1016/s0161-6420(83)34542-5

[4] Hayreh SS. Neovascular glaucoma. Prog Retin Eye Res. 2007;26: 470–485. 10.1016/j.preteyeres.2007.06.001 17690002 PMC2871536

[5] McIntosh RL, Rogers SL, Lim L, Cheung N, Wang JJ, Mitchell P, et al Natural history of central retinal vein occlusion: an evidence-based systematic review. Ophthalmology. 2010;117: 1113–1123.e15. 10.1016/j.ophtha.2010.01.060 20430446

[6] Joussen AM, Gardner TW, Kirchhof B, Ryan SJ. Retinal vascular disease. Heidelberg: Springer Science Buisness Media; 2007.

[7] Hayreh SS, Zimmerman MB. Ocular neovascularization associated with central and hemicentral retinal vein occlusion. Retina. 2012;32: 1553–1565. 10.1097/IAE.0b013e318246912c 22495331

[8] Rogers SL, McIntosh RL, Lim L, Mitchell P, Cheung N, Kowalski JW, et al Natural history of branch retinal vein occlusion: an evidence-based systematic review. Ophthalmology. 2010;117: 1094–1101.e5. 10.1016/j.ophtha.2010.01.058 20430447

[9] Shilling JS, Kohner EM. New vessel formation in retinal branch vein occlusion. Br J Ophthalmol. 1976;60: 810–815. 1009063 10.1136/bjo.60.12.810 PMC1042850

[10] Branch Vein Occlusion Study Group: Argon laser scatter photocoagulation for prevention of neovascularization and vitreous hemorrhage in branch vein occlusion. A randomized clinical trial. Branch Vein Occlusion Study Group. Arch Ophthalmol. 1986;104: 34–41. 2417579 10.1001/archopht.1986.01050130044017

[11] Butler JM. Role of stromal cell-derived factor-1 in proliferative retinopathy. A dissertation presented to the graduate school of the university of Florida in partial fulfillment of the requirements for the degree of doctor of philosphy. 2006. Available: http://ufdcimages.uflib.ufl.edu/UF/E0/01/33/94/00001/butler_j.pdf

[12] Butler JM, Guthrie SM, Koc M, Afzal A, Caballero S, Brooks HL, et al SDF-1 is both necessary and sufficient to promote proliferative retinopathy. J Clin Invest. 2005;115: 86–93. 10.1172/JCI22869 15630447 PMC539202

[13] Aiuti A, Webb IJ., Bleul C, Springer T, Gutierrez-Ramos JC. The chemokine SDF-1 is a chemoattractant for human CD34+ hematopoietic progenitor cells and provideds a new mechanism to explain the mobilization of CD34+ progenitors to peripheral blood. J Exp Med. 1997;185: 111–120. 8996247 10.1084/jem.185.1.111 PMC2196104

[14] Salcedo R, Zhang X, Young HA, Michael N, Wasserman K, Ma WH, et al Vascular endothial growth factor and basic fibroblast growth factor induce expression of CXCR4 on human endothelial cells: in vivo neovascularization induced by stromal-derived factor-1. Am J Pathol. 1999;154: 1125–1135. 10233851 10.1016/s0002-9440(10)65365-5 PMC1866563

[15] Ki-I Y, Arimura N, Noda Y, Yamakiri K, Doi N, Hashiguchi T, et al Stromal-derived factor-1 and inflammatory cytokines in retinal vein occlusion. Curr Eye Res. 2007;32: 1065–1072. 10.1080/02713680701758727 18085471

[16] Colobran R, Pujol-Borrell R, Armengol MP, Juan M. The chemokine network. II. On how polymorphisms and alternative splicing increase the number of molecular species and configure intricate patterns of disease susceptibility. Clinical and Experimental Immunology. 2007;150: 1–12. 10.1111/j.1365-2249.2007.03489.x 17848170 PMC2219280

[17] Luan B, Han Y, Zhang X, Kang J, Yan C. Association of the SDF1-3′A polymorphism with susceptibility to myocardial infarction in Chinese Han population. Molecular Biology Reports. 2010;37: 399–403. 10.1007/s11033-009-9845-3 19821058

[18] Gu XL, Ma N, Xiang DC, Huang J, Dong ZH, Lei HY, et al Polymorphism of stromal cell-derived factor-1 selectively upregulates gene expression and is associated with increased susceptibility to coronary artery disease. Biochem Biophys Res Commun. 2014;443: 932–937. 10.1016/j.bbrc.2013.12.065 24361877

[19] Lam FC, Chia SN, Lee RM. Macular grid laser photocoagulation for branch retinal vein occlusion. Cochrane Database Syst Rev. 2015;11: CD008732 10.1002/14651858.CD008732.pub2 25961835 PMC10879914

[20] Glass TJ, Lund TC, Patrinostro X, Tolar J, Bowman TV, Zon LI, et al Stromal cell-derived factor-1 and hematopoietic cell homing in an adult zebrafish model of hematopoietic cell transplantation. Blood. 2011;118: 766–774. 10.1182/blood-2011-01-328476 21622651 PMC3292438

[21] Sahin AO, Buitenhuis M. Molecular mechanisms underlying adhesion and migration of hematopoietic stem cells. Cell Adh Migr. 2012;6: 39–48. 10.4161/cam.18975 22647939 PMC3364136

[22] Lima e Silva R, Shen J, Hackett SF, Kachi S, Akiyama H, Kiuchi K, et al The SDF-1/CXCR4 ligand/receptor pair is an important contributor to several types of ocular neovascularization. FASEB J. 2007;21: 3219–3230. 10.1096/fj.06-7359com 17522382

[23] Abu El-Asrar AM, Struyf S, Kangave D, Geboes K, Van Damme J. Chemokines in proliferative diabetic retinopathy and proliferative vitreoretinopathy. Eur Cytokine Netw. 2006;17: 155–165. 17194635

[24] Kijowski J, Sagi-Assif O, Meshel T, Geminder H, Goldberg-Bittman L, Ben-Menachem S, et al The SDF-1-CXCR4 axis stimulates VEGF secretion and activates integrins but does not affect proliferation and survival in lymphohematopoietic. Stem Cells. 2001;19: 453–466. 10.1634/stemcells.19-5-453 11553854

[25] Burger M, Glodek A, Hartmann T, Schmitt-Gräff A, Silberstein LE, Fujii N, et al Functional expression of CXCR4 (CD184) on small-cell lung cancer cells mediates migration, integrin activation, and adhesion to stromal cells. Oncogene. 2003;22: 8093–8101. 10.1038/sj.onc.1207097 14603250

[26] Cardones AR, Murakami T, Hwang ST. CXCR4 enhances adhesion of B16 tumor cells to endothelial cells in vitro and in vivo via beta(1) integrin. Cancer Res. 2003;63: 6751–6757. 14583470

[27] Raffi S, Hessig B, Hattori K. Efficient mobilization and recruitment of marrow-derived endothelial and hematopoietic stem cells by adenoviral vectors expressing angiogenic factors. Gene Ther. 2002;9: 631–641. 10.1038/sj.gt.3301723 12032709

[28] Chen LY, Zhuo YH, Li YH, Huang XH, Zhang JL, Li SY, et al Expression of stromal cell-derived factor-1 in diabetic retinopathy Chinese Medical Journal. 2010;123: 984–988. 20497701

[29] Sonmez K, Drenser KA, Capone A Jr, Trese MT. Vitreous levels of stromal cell-derived factor 1 and vascular endothelial growth factor in patients with retinopathy of prematurity. Ophthalmology. 2008;115: 1065–1070.e1. 10.1016/j.ophtha.2007.08.050 18031819

[30] Djuric Z, Sharei V, Rudofsky G, Morcos M, Li H, Hammes HP, et al Association of homozygous SDF-1 3'A genotype with proliferative diabetic retinopathy. Acta Diabetol. 2010;47: 79–82. 10.1007/s00592-009-0119-2 19381432

[31] Ide A, Kawasaki E, Abiru N, Sun F, Fukushima T, Takahashi R, et al Stromal-cell derived factor-1 chemokine gene variant is associated with type 1 diabetes age at onset in Japanese population. Hum Immunol. 2003;64: 973–978. 14522095 10.1016/s0198-8859(03)00176-9

[32] Warchol T, Lianeri M, Lacki JK, Jagodziński PP. SDF1-3' G801A polymorphisms in Polish patients with systemic lupus erythematosus. Mol Biol Rep. 2010;37: 3121–3125. 10.1007/s11033-009-9890-y. Epub 2009 Oct 14. 19826912

[33] Dubois-Laforgue D, Hendel H, Caillat-Zucman S, Zagury JF, Winkler C, Boitard C, et. al A common stromal cell-derived factor-1 chemokine gene variant is associated with the early onset of type 1 diabetes. Diabetes. 2001;50: 1211–1213. 11334429 10.2337/diabetes.50.5.1211

[34] The Eye Disorders Case-Control Study Group Risk Factors for Central retinal vein occlusion. Arch Ophthalmol. 1996;114: 545–554. 8619763

[35] Santiago JG, Sun JK, Silva PS, Haddad ZA, Aiello LP. Risk Factors for Intraocular Ischemia and Neovascularization in Central Retinal Vein Occlusion (CRVO) in Diabetic versus Nondiabetic Patients. Investigative Ophthalmology & Visual Science. 2009; 50: 3720.

[36] Funderburk RL, Feinberg EB. Diabetes as a risk factor for retinal neovascularization in retinal vein occlusion. Ann Ophthalmol. 1989;21: 65–66. 2469374

